# Weakly acidic microenvironment of the wound bed boosting the efficacy of acidic fibroblast growth factor to promote skin regeneration

**DOI:** 10.3389/fbioe.2023.1150819

**Published:** 2023-03-01

**Authors:** Qiao Pan, Ruyi Fan, Rui Chen, Jiayi Yuan, Shixuan Chen, Biao Cheng

**Affiliations:** ^1^ The First School of Clinical Medicine, Southern Medical University, Guangzhou, China; ^2^ Department of Burn and Plastic Surgery, General Hospital of Southern Theater Command of PLA, Guangzhou, China; ^3^ Zhejiang Engineering Research Center for Tissue Repair Materials, Wenzhou Institute, University of Chinese Academy of Sciences, Wenzhou, Zhejiang, China; ^4^ School of Basic Medical Sciences, Southern Medical University, Guangzhou, China

**Keywords:** wound healing, fibroblast growth factor, microenvironment, pH value, inflammation

## Abstract

The pH value within the wound microenvironment influences indirectly and directly all biochemical reactions taking place in the process of skin wound healing. Currently, it is generally believed that a low pH value, such as it is found on normal skin, is favorable for wound regeneration, while some investigations have shown that in fact alkaline microenvironments are required for some healing processes. The role of growth factors in promoting wound healing requires a specific microenvironment. In wound microenvironments of different pH, growth factors with different isoelectric points may have different effects. To explore whether the application of FGF with different isoelectric points in wounds with different pH values interferes with the healing process to different degrees, GelMA hydrogels with different pH values were prepared to maintain the wounds microenvironment with the same pH values, in which aFGF and bFGF were loaded as well. The results show that GelMA hydrogels of different pH values maintained the same pH of the wound microenvironment sustainably on the 4th day. Moreover, aFGF and bFGF promoted skin wound healing to varying degrees in different pH wound microenvironments. In particular, aFGF significantly promoted wound re-epithelialization in a weak acidic microenvironment, while bFGF promoted collagen synthesis and deposition in the early stage of weak acid wounds. In addition, aFGF plays a superior role in inhibiting inflammation in weak acidic wounds.

## 1 Introduction

Skin wound healing is a dynamic and sequential process with multiple events, including the secretion and release of cytokines, and various cell activities (*e.g.*, proliferation, migration, differentiation, as well as the synthesis and remodeling of extracellular matrix) (Gillitzer and Goebeler, 2001; Gurtner et al., 2008). During this process, the pH value of the local microenvironment is a very important factor that can indirectly or directly affect a series of repair reactions during the process of wound healing (Schneider et al., 2007; Schreml et al., 2010). The pH value of normal skin is between 4.8 and 6.0 due to the organic acid secreted by keratinocytes. Bennison et al. (2017) When the integrity of the skin is damaged, alkaline tissue fluid and the plasma in the broken capillaries are extravasated, the pH value of the wound area will significantly increase. Milne and Connolly (2014) Previous studies have shown that a low pH value of the wound microenvironment is more conducive to wound healing, especially when it is closer to normal skin. Sim et al. (2022) Under the acidic microenvironment of the wound, not only the infection risk is low, but also the granulation tissue formation and neovascularization are effectively promoted. While in the alkaline wound microenvironment, bacterial infection and biofilm are more easily formed. In addition, protease hydrolysis is further intensified, especially in a chronic wound, which is harmful to wound healing.

Growth factors, including fibroblast growth factor (FGF), platelet-derived growth factor-BB (PDGF-BB), Epidermal growth factor (EGF), Vascular endothelial cell growth factor (VEGF), and so on are essential to wound healing (Barrientos et al., 2008; Schultz and Wysocki, 2009; Behm et al., 2012). Among these growth factors, FGFs are not only the most common mitogens, but also are multifunctional growth factors (Turner and Grose, 2010; Ornitz and Itoh, 2015). In terms of wound healing, FGFs are all-powerful growth factors. It is capable of regulating several important biological events of wound healing, such as granulation tissue formation, angiogenesis, and re-epithelialization (Hui et al., 2018; Guo et al., 2019). In humans, 23 members of the FGF family have been identified, Ornitz and Itoh (2001) and FGF1 and FGF2 are the most common to know. The FGF1 is also known as the acidic fibroblast growth factor (aFGF), and FGF2 is also known as the basic fibroblast growth factor (bFGF). Both the aFGF and bFGF have been widely encapsulated into hydrogels, particles and nano/micro-fibers for the application of acute and chronic wound repair. However, these studies did not consider the influence of local pH value of wounds and substrates. The changes in pH value of the microenvironment may affect the effectiveness of growth factors with different isoelectric points.

Herein, we hypothesized that aFGF or bFGF with different isoelectric points applied to the wound bed with different pH may interfere with the healing process to varying degrees. GelMA hydrogel is widely used in various drug release systems and wound biological dressing materials due to its suitable biological properties, adjustable physical properties, and basic characteristics that are similar to the natural extracellular matrix. To verify the above hypothesis, GelMA hydrogel was selected as the carrier of two growth factors to extend their action time, we also adjusted the pH value of the hydrogel to regulate the pH value of the wound microenvironment, so that to observe the healing role of aFGF and bFGF in different microenvironments during the process of wound healing.

## 2 Materials and methods

### 2.1 Preparation of acidic- and basic- FGF-loaded GelMA hydrogels with different pH values

GelMA was dissolved in phosphate-buffered saline (PBS) to prepare GelMA solution at a certain concentration of 6% (w/v). The solution was stirred in a water bath at 37 °C and 500 r/min until the GelMA solid completely dissolved. All GelMA solutions contained 2,959 (Irgacure2959, Sigma-Aldrich, United States , 98% pure) at a concentration of 0.5% (w/v), and the pH value of the solution was adjusted to 6.4, 7.4, and 8.4, respectively. The aFGFs and bFGFs were separately added to the GelMA solutions with different pH values at a concentration of 4ug/ml. Then solutions of 6.4-GelMA/aFGF, 7.4-GelMA/aFGF, 8.4-GelMA/aFGF, 6.4-GelMA/bFGF, 7.4-GelMA/bFGF, and 8.4-GelMA/bFGF were sterilely filtered through a 25 µm microporous membrane. 50μl of GelMA solution was placed into each cylindrical mold with an inner diameter of 8 mm and a height of 1 mm and each hydrogel scaffold containing 200 ng of FGF was subsequently fabricated by photo cross-linking.

### 2.2 SEM observation

The internal structure of the freeze-dried GelMA hydrogels was observed using a scanning electron microscope (Hitachi S-3400N, Japan). Before observation, platinum was coated by ion sputtering (Hitachi E−1010) for 60 seconds. Before taking digital photomicrographs from randomly selected areas of the samples with a magnification of ×100 and 400×, at an acceleration voltage of 5 kV.

### 2.3 Swelling ratio test

The swelling behaviors of GelMA hydrogel with different pH values were measured by weighing both the initial and swollen hydrogels. Briefly, the initial weights of the hydrogel scaffolds (W0 values) were measured. The hydrogel scaffolds were then placed in PBS (Gibco, United States ) at 37 °C and allowed to swell for 15, 30, 45, 60, 90, 120, and 180 min. The wet weights of each of the hydrogel scaffolds (Wt values) were measured at each time point. The swelling ratios were calculated using the equation below. For this test, n = 10, and each measurement was repeated 3 times.

$$Esr\;\left(\%\right)=\left(Wt\;–W0\right)/W0\times 100\%$$

Here, Esr (%) is the swelling rate of the hydrogel, Wt is the wet weight of the hydrogel after absorbing water, and W0 is the initial dry weight of the hydrogel.

### 2.4 Drug release test

The release test of aFGFs and bFGFs from GelMA hydrogels with different pH values was performed. Briefly, the hydrogel was placed in 2 ml PBS in a 37 °C incubator and 50 ul of PBS was replaced by the same volume of fresh PBS at distinct time points (1,3,5,9,14 days). The concentration of aFGF and bFGF in removed PBS was measured by the ELISA Kit (Bioswamp) and Microplate Reader at 450 nm. For this test, n = 4, and each measurement was repeated 3 times.

### 2.5 Wound healing

Eight-week-old specific-pathogen-free (SPF)-class female C57BL/6 mice were purchased from the Zhejiang Laboratory Animal Center. All animal procedures were approved by the Institutional Animal Care and Use Committee (IACUC) at Wenzhou Institute, University of Chinese Academy of Sciences. For wounding, the mice were anesthetized with isoflurane. The hair was removed from the dorsal region, and the skin was prepared with betadine and 70% ethanol. A skin biopsy punch was used to make a circular full-thickness skin wound with a diameter of 8 mm on both sides of the dorsal skin. A splint was spread with an instant bonding adhesive and placed around the wound and secured to the skin with eight interrupted 6–0 nylon sutures.

The post-surgery mice were randomly assigned to one of seven groups of three mice each, treated with 6.4-GelMA/aFGF, 7.4-GelMA/aFGF, 8.4-GelMA/aFGF, 6.4-GelMA/bFGF, 7.4-GelMA/bFGF, 8.4-GelMA/bFGF hydrogel scaffold, and no hydrogel scaffold. Scaffolds were attached to the wound site immediately after the wounding. The wounds and splints were covered with Tegaderm (3M) sterile transparent dressing. A digital camera was used to record the wounds on each mouse on days 0, 4, 8 and 12 after treatment. The wound-healing rates were calculated by the following equation: wound-healing rate = (the area of the original wound - the area of the unhealed wound)/the area of the original wound 100%.

### 2.6 Histological analysis

On postoperative days 4, 8, and 12, mice were sacrificed and a complete wound with a 0.5 cm margin was removed. After being fixed in 4% paraformaldehyde solution (PFA) for 2 days, the samples were rinsed in PBS, 50%, 70%, 80%, 90%, 95%, and 100% ethanol solution for gradient dehydration. After xylene clear treatment and gradient wax dipping, embedded samples in paraffin to make 5-μm sections for hematoxylin and eosin (H&E) staining. The wound re-epithelialization rate was calculated by the following formula: Rate of re-epithelialization (%) = St/S0 100%

S0 is the initial wound distance for the section and St is the keratinocytes covering the wound distance at the indicated time for that section. Masson’s trichrome staining was performed by Masson’s trichrome staining kit (Cat# MST-8004, MaiXin-Bio, China) according to the manufacturer’s instructions.

### 2.7 Immunohistochemical staining

Deparaffinized tissue sections were heated in citrate buffer to retrieve the antigens and then treated with 3% H2O2 for 15 min to inactivate endogenous peroxidase activity. Sections were then blocked with goat serum and BSA solution for 1 h and incubated with primary antibodies overnight at 4 °C. The primary antibodies included α-SMA (Boster, China, Catalog#BM0002), CD31(Proteintech, China, Catalog#28083-1-AP), CCR7 (Abcam, United States ; Catalog#EPR23192-57), and CD206 (Abcam, United States ; Catalog#EPR25215-277). The next day, after being treated with biotinylated secondary antibodies for 1 h at room temperature, the peroxidase activity of tissue section was determined with diaminobenzidine (DAB). Image-Pro Plus was used to analyze the average numbers of the positive cells for α-SMA, CD31, CCR7, and CD206 expression.

### 2.8 Statistical analysis

Data are presented as mean ± standard error of the mean. Statistical significance was determined by one-way analysis of variance (ANOVA) followed by Tukey’s posttest using GraphPad Prism software (version 8.0). A value of *p* < 0.05 was considered statistically significant (significance levels: *p < 0.05, and **p < 0.01).

## 3 Results and discussion

### 3.1 Preparation of GelMA hydrogels with different pH values

**FIGURE 1:**
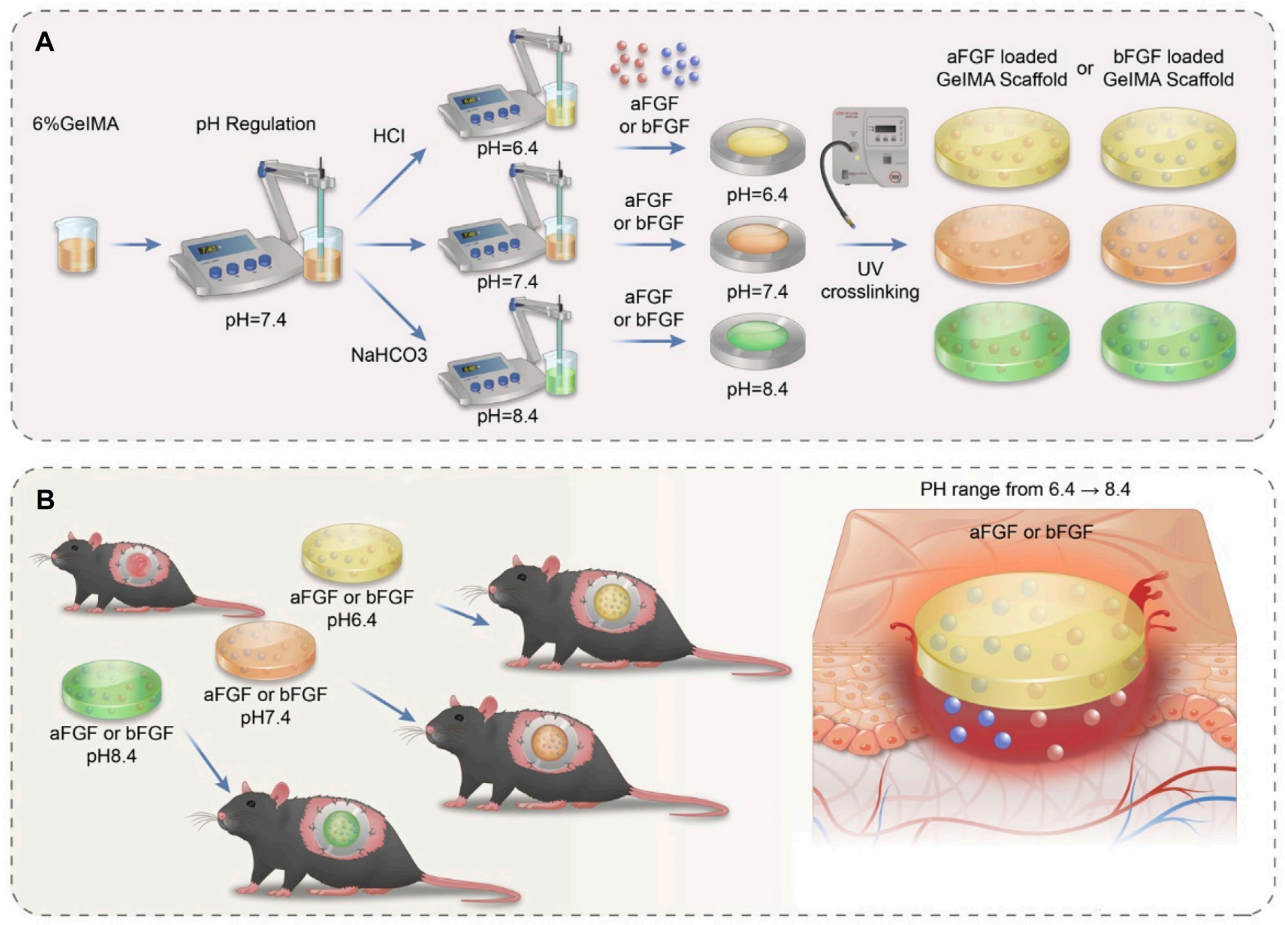
The schematic illustrates the preparation processes and their application on skin wound healing of acidic- and basic-fibroblast growth factors loaded GelMA hydrogels with different pH values. **(A)** The preparation processes of acidic- and basic-fibroblast growth factors loaded GelMA hydrogels with different pH values. The pH value of GelMA hydrogel was precisely controlled by adding hydrochloric acid and sodium bicarbonate **(B)** The application of acidic- and basic-fibroblast growth factors loaded GelMA hydrogels with different pH values on skin wound healing.

**FIGURE 2:**
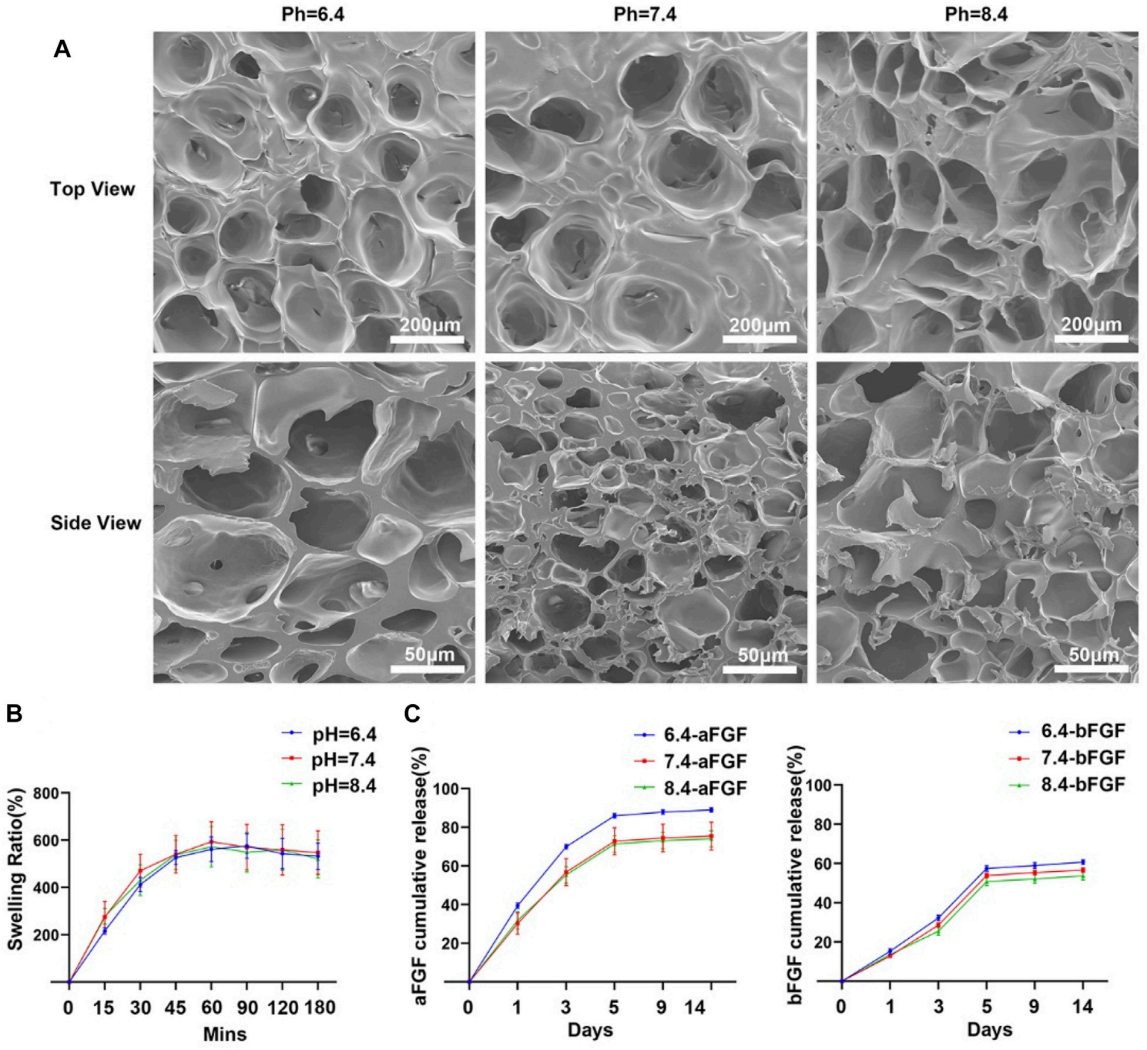
The internal structure and release profiles of aFGFs and bFGFs loaded GelMA hydrogels with different pH values. **(A)** The internal porous structure of GelMA hydrogels with different pH values. **(B)** The swelling behaviors of GelMA hydrogel with different pH value. **(C)** The release profiles of aFGFs and bFGFs from GelMA hydrogels with different pH values.

GelMA is an widely used biomaterials with excellent biocompatibility in the field of tissue regeneration (Yue et al., 2015). Thus, we choose the GelMA hydrogel as a substrate. As shown in Figure 1A, hydrochloric acid and sodium bicarbonate were used to adjust the pH values of GelMA hydrogels to 6.4, 7.4 and 8.4 respectively. Then the aFGF and bFGF were mixed into hydrogel solution, and hydrogels were crosslinked by UV irradiation. In this study, the effects of the micro environment created by GelMA hydrogel with different pH values on the efficacy of aFGF or bFGF were explored in a skin wound healing model (Figure 1B). The GelMA hydrogels with different pH values showed a porous structure, and there was no difference on the pore size among the three different hydrogels (Figure 2A), resulting in these hydrogels showed a similar swelling behaviors (Figure 2B), the final swelling rate of the GelMA hydrogels with different pH values range from 6.4 to 8.4 were (532.029 ± 55.869)% (547.569 ± 92.310)%, and (521.103 ± 79.775)% respectively. The release curves discovered that the release of aFGF from weakly acidic GelMA hydrogel (pH 6.4) was faster than it released from neutral (pH 7.4) and weakly alkaline (pH 8.4) GelMA hydrogels (Figure 2C). The cumulative release of aFGF from the pH 6.4 GelMA hydrogel ((88.965 ± 1.157) %) was higher than it released from the pH 7.4 GelMA hydrogel ((75.465 ± 7.151) %) and pH 8.4 GelMA hydrogel ((74.068 ± 4.228) %). There was no difference on the release of bFGF from weakly acidic, neutral and weakly alkaline GelMA hydrogels (Figure 2C).

### 3.2 aFGF loaded GelMA hydrogel (pH 6.4) promotes wound contraction

**FIGURE 3:**
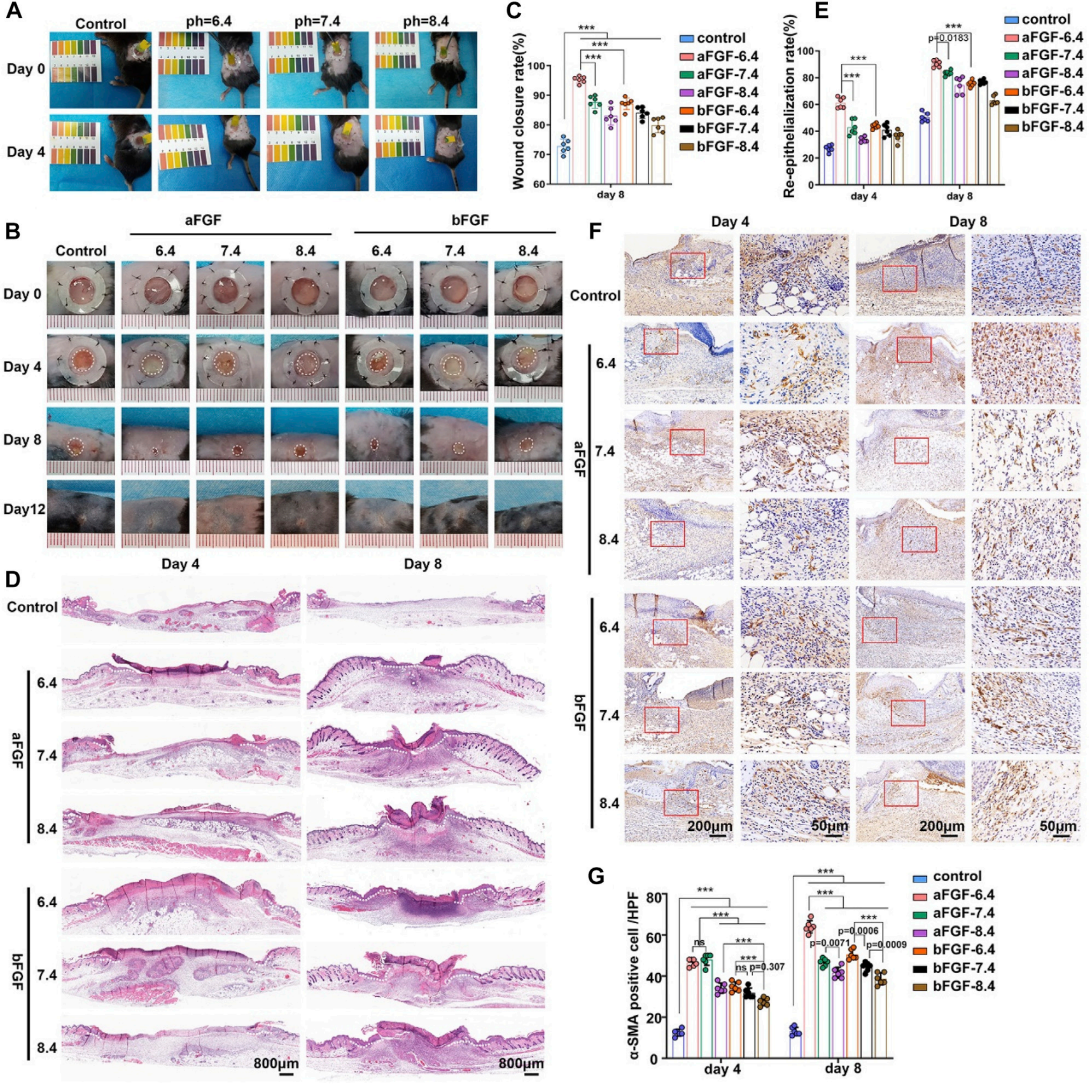
The application of aFGF- or bFGF- loaded GelMA hydrogels with different pH values on wound closure. **(A)** The maintenance of weak acid, neutral, and alkaline microenvironments of wound sites filled with GelMA hydrogels with different pH values. **(B)** The photographs and H&E staining of the healed wound treated with aFGF- or bFGF- loaded GelMA hydrogels with different pH values for 8 days. **(C)** The wound closure rate of aFGF- or bFGF- loaded GelMA hydrogels with different pH values after 4 and 8 days of treatment. **(D)** The H&E staining results of aFGF- or bFGF- loaded GelMA hydrogels with different pH values after 4 and 8 days of treatment. **(E)** The re-epithelialization rate of aFGF- or bFGF- loaded GelMA hydrogels with different pH values after 4 and 8 days of treatment. **(F,G)** The expression of α-SMA within the wound area of aFGF- or bFGF- loaded GelMA hydrogels with different pH values after 4 and 8 days of treatment. *p < 0.05, **p < 0.01, ***p < 0.001.

The purpose of this study is to explore the effects of the micro environment created by GelMA hydrogel with different pH values on the efficacy of aFGF or bFGF were explored in a skin wound healing model. Thus, the pH value of micro environment created by the hydrogel is very important. Firstly, we examined whether the GelMA hydrogels with different pH values are able to control the desirable pH values. As shown in Figure 3A, the GelMA hydrogel (pH 6.4) could maintain a weakly acidic environment, the GelMA hydrogel (pH 7.4) could maintain a weakly acidic environment, the GelMA hydrogel (pH 7.4) could maintain a neutral environment, and GelMA hydrogel (pH 8.4) could maintain a weakly alkaline environment. We speculate that the maintenance of local pH values of wound site is achieved by the sustained release of hydrochloric acid and sodium bicarbonate from GelMA hydrogel. After applying the aFGF or bFGF loaded GelMA hydrogels with different pH value, we found the wounds treated with aFGF loaded GelMA hydrogel (pH 6.4) exhibited fastest wound closure rate and achieved wound healing on Day 8 (Figures 3B,C). The H&E staining further revealed that the wounds treated with aFGF loaded GelMA hydrogel (pH 6.4) also showed a fastest re-epithelialization rate when compared with other groups on both Day 4 and Day 8 (Figure 3B). Wound contraction is drove by the myofibroblasts, which highly expressed α-SMA. The immunohistochemical staining results discovered that the expression of α-SMA of the wounds treated with aFGF loaded GelMA hydrogel (pH 6.4) was significantly increased than the other groups (Figures 3E,F). According to the reported studies, both aFGF and bFGF were all able to promote wound contraction (Wu et al., 2016; Choi et al., 2018; Hui et al., 2018). However, there are few reports on the effect of the pH values of the micro environment on FGF activity. In this study, we found weakly acidic environment is more conducive to the function of aFGF. It may be associated with the physical stabilization of aFGF which induced by the weakly acidic environment (Volkin et al., 1993).

### 3.3 aFGF loaded GelMA hydrogel (pH 6.4) accelerates angiogenesis

**FIGURE 4:**
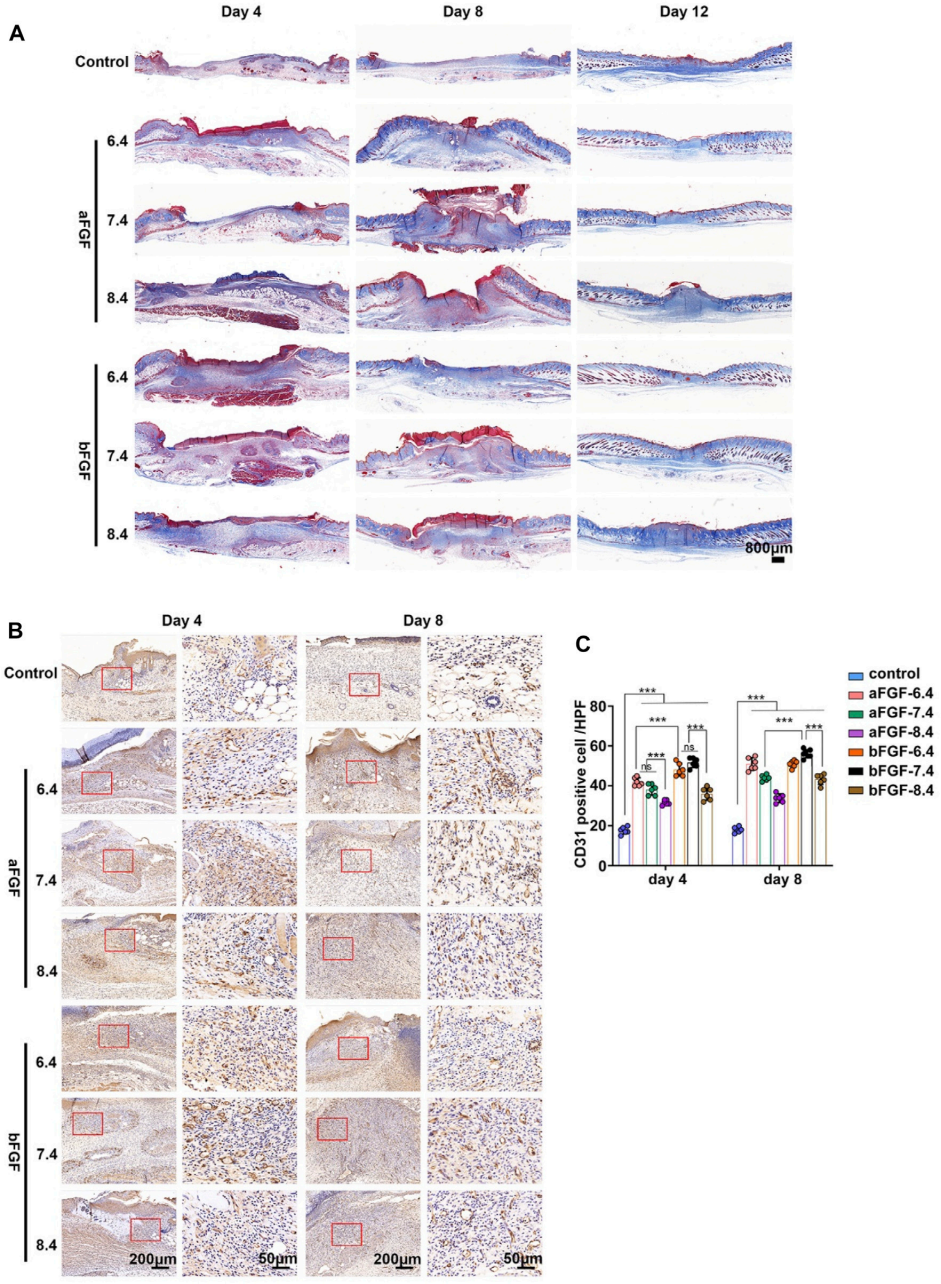
The granulation tissue formation of the wounds treated with aFGF- or bFGF- loaded GelMA hydrogels with different pH values for 4 and 8 days. **(A)** The trichrome staining of the wounds treated with aFGF- or bFGF- loaded GelMA hydrogels with different pH values for 4, 8, and 12 days. **(B)** The immunohistochemical staining of α-SMA within the wounds treated with aFGF- or bFGF- loaded GelMA hydrogels with different pH values for 4 and 8 days. **(C)** The quantification of the α-SMA expression of the **(B)**. *p < 0.05, **p < 0.01, ***p < 0.001.

Vascularization plays important role during wound healing, it can provide enough nutrients for cells involved in wound repair (Wan et al., 2019; Chen et al., 2020). As shown in Figure 4A, the trichrome staining results showed lots of new blood vessels in the wound bed of aFGF loaded GelMA hydrogel (pH 6.4) when compared with six groups on Day 4. These new blood vessels provide enough nutrients in the early stage of wound healing, resulting in accelerating wound healing. The number of blood vessels of wound site will gradually decrease after wound closure on Day 8. CD31 is a specific marker of vascular endothelial cells (Sun et al., 2011). The CD31 immunohistochemical staining further confirmed the histological observations. The expression of CD31 on aFGF loaded GelMA hydrogel (pH 6.4) treated wound was higher than the other six groups on Day 4, and the expression of CD31 in aFGF loaded GelMA hydrogel (pH 6.4) treated group on Day 8 was reduced than it on Day 4 (Figure 4B; 4C).

### 3.4 aFGF loaded GelMA hydrogel (pH 6.4) regulates the immune responses to a pro-regenerative status

**FIGURE 5:**
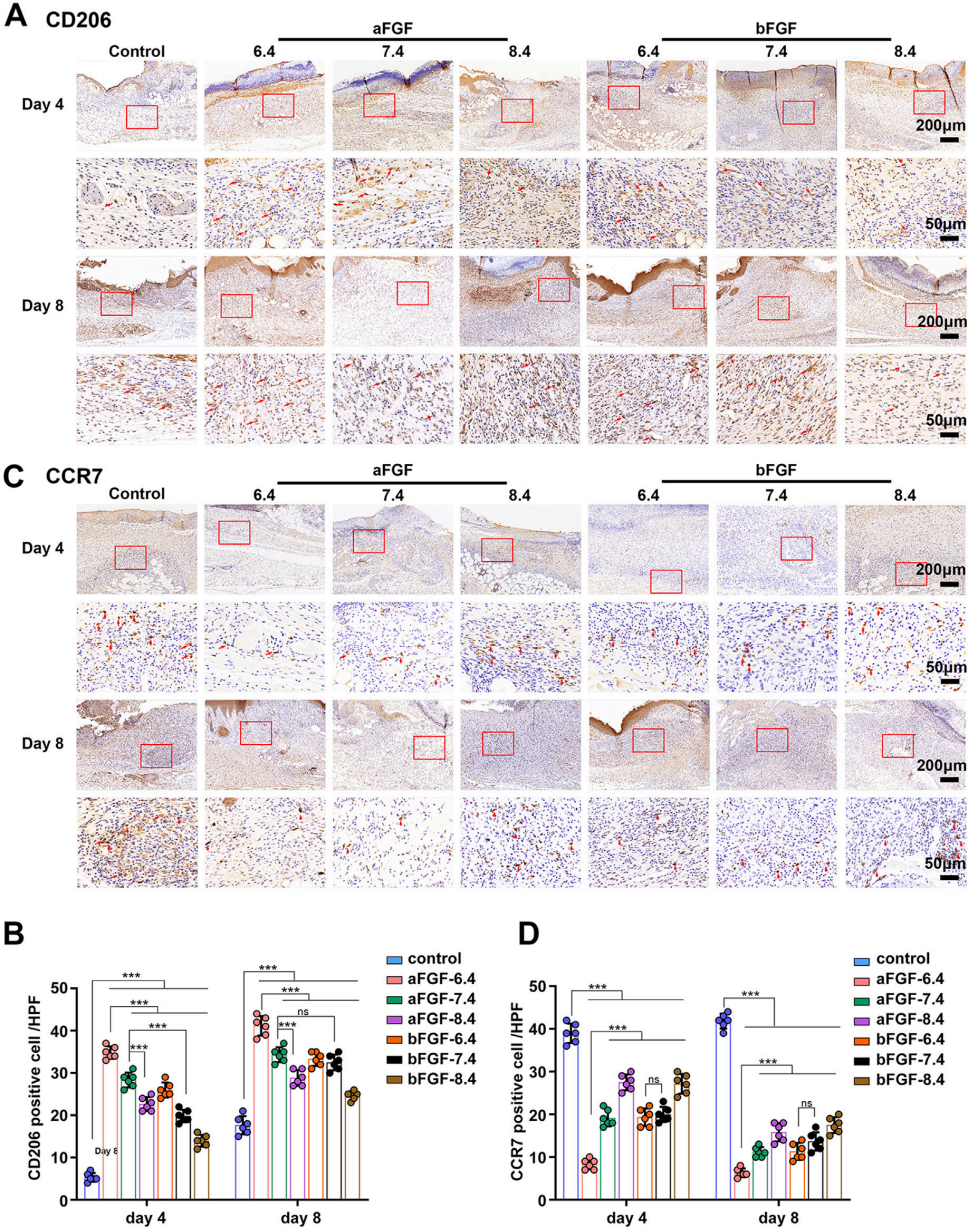
The expression of macrophages within the wounds treated with aFGF- or bFGF- loaded GelMA hydrogels with different pH values for 4 and 8 days. **(A,B)** The expression of CD206 (M2 macrophages) within the wounds treated with aFGF- or bFGF- loaded GelMA hydrogels with different pH values for 4 and 8 days. **(C,D)** The expression of CCR7 (M1 macrophages) within the wounds treated with aFGF- or bFGF- loaded GelMA hydrogels with different pH values for 4 and 8 days.

Immune response is capable of determining the status of wound healing (Jiang et al., 2018; Chen et al., 2019). For example, if the inflammatory response of the wound is in a pro-inflammatory state, it is harmful to wound healing, such as diabetic foot ulcers. If the inflammatory response of the wound is in a state of pro-regenerative status, it will help to promote wound healing. The status of inflammation depends on the involved inflammatory cells. Macrophages are the main cells of the inflammatory response and play a major role in controlling the entire inflammatory process (Kim and Nair, 2019). As shown in Figures 5A,C, the expression of CD206 (a marker of M2 macrophages) of the wounds treated with aFGF loaded GelMA hydrogel (pH 6.4) was significantly higher than the other six groups on Day 4. While the expression of CCR7 (a marker of M1 macrophages) of the wounds treated with aFGF loaded GelMA hydrogel (pH 6.4) was obvious lower than the other six groups on Day 4 (Figures 5B,D). M2 macrophages play pro-regenerative role, while M1 macrophages play pro-inflammatory role. The increased M2 macrophage expression and decreased M1 macrophage expression co-determined the direction of inflammatory response is pro-regenerative status (Jiang et al., 2018; Chen et al., 2019). In addition, the expression of CD206 in aFGF loaded GelMA hydrogel (pH 6.4) treated group was reduced when compared its expression on Day 4. Because the wounds already completed wound healing, the status of wound healing has changed from proliferative phase to remodeling phase that the role of macrophages go down.

### 3.5 The exploration of potential mechanism

**FIGURE 6:**
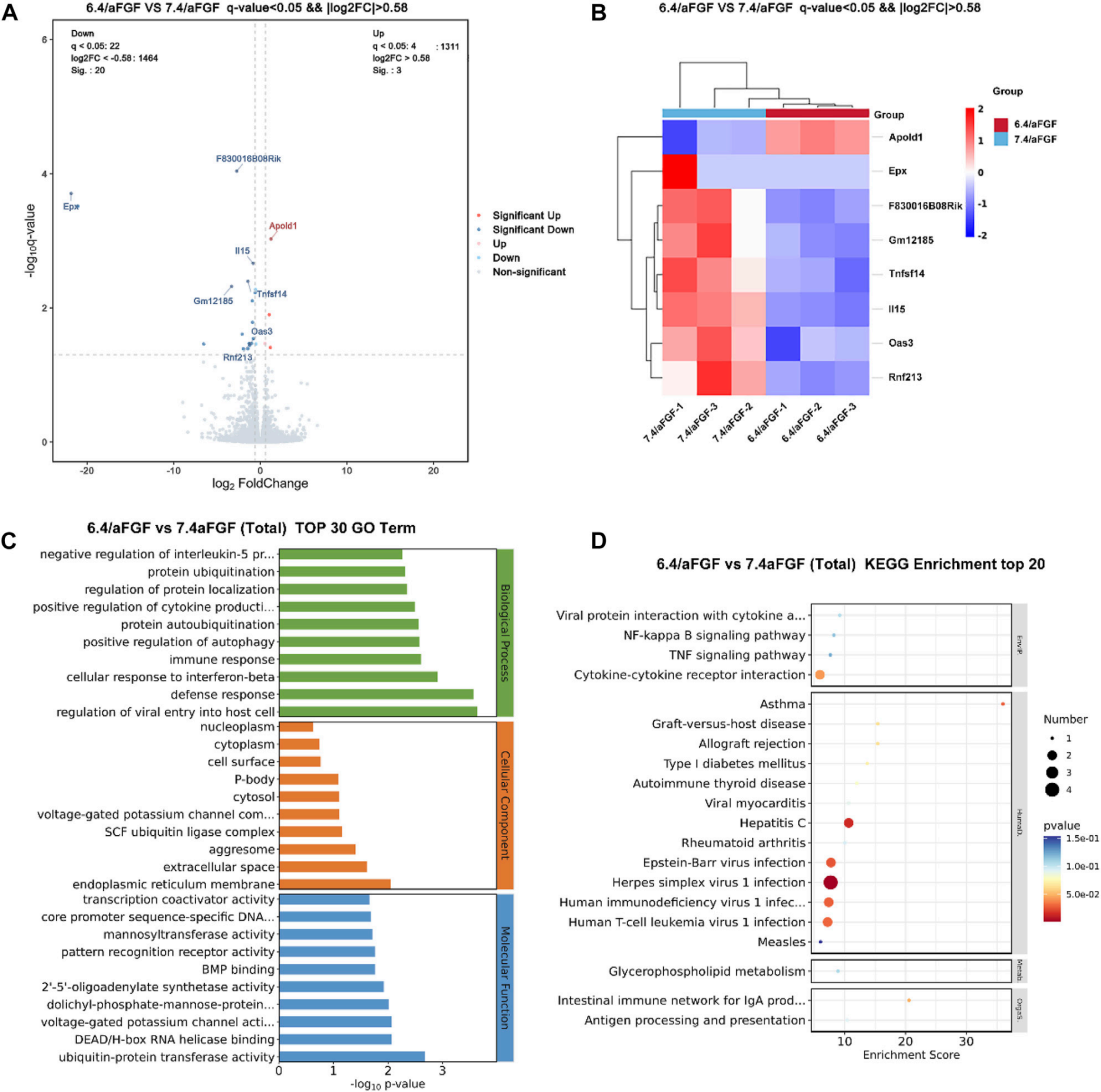
RNA-seq analysis of differentially expressed genes and signaling pathways of the wounds treated with aFGF loaded GelMA hydrogel with 6.4 and 7.4 pH value. **(A,B)** Volcano plot and heatmap discover the differentially expressed genes. **(C,D)** The potential signaling pathway involve in the wound healing processes.

As shown in Figure 6, The volcano plot and heatmap discover the differentially expressed genes (Figures 6A,B), including Apold1, Epx, F830016B08Rik, Gm12185, Tnfsf14, il15, Oas3, Rnf213. For example, Apold1 is an endothelial cell early response protein that may play a role in regulation of endothelial cell signaling and vascular function (Stritt et al., 2022). The upregulation of Apold1 can promote angiogenesis, which is consistent with the results of trichrome staining and the expression of CD31. The residual down-expressed genes, including Epx, il15, Oas3, Tnfsf14 were able to regulate the immune responses. It is consistent with the increased expression of M2 macrophages and decreased expression of M1 macrophages. In addition, the significant enrichment analysis of GO function revealed that protein ubiquitination, regulation of protein localization, positive regulation of cytokine production and immune responses (Figure 6C). The KEGG enriched signaling pathway analysis discovered the immune regulation related signaling pathways, such as NF-kappa B signaling pathway and TNF signaling pathway, were the most significant (Figure 6D). Immunomodulation plays critical role during the wound healing, Chen et al. (2023) thus the improved immune response is capable of accelerating wound healing.

## 4 Conclusion

In this study, we have tried to explored the influence of the local pH value of wound site on efficacy of fibroblast growth factor. We found aFGF and bFGF played similar role on promoting wound healing under neutral and alkaline conditions. However, aFGF exhibited significant promoting effects than the bFGF when the local pH value of wound site was adjusted to acidic conditions. The local acidic pH value helped fibroblast growth factors to play significant role in promoting wound contraction, granulation tissue formation, vascularization, re-epithelialization.

## Data Availability

The RNA-seq data presented in the study are deposited in the GEO repository, accession number GSE225505.
